# Melatonin Sources in Sheep Rumen and Its Role in Reproductive Physiology

**DOI:** 10.3390/ani14233451

**Published:** 2024-11-28

**Authors:** Tian Niu, Ziqiang Ding, Jianlin Zeng, Zhenxing Yan, Hongwei Duan, Jianshu Lv, Yong Zhang, Lihong Zhang, Junjie Hu

**Affiliations:** 1 College of Veterinary Medicine, Gansu Agricultural University, Lanzhou 730070, China; nt18393170049@163.com (T.N.); zengjl0123@163.com (J.Z.); yzx17693397404@163.com (Z.Y.); grand6138@163.com (H.D.); lvjs153@163.com (J.L.); zhychy@163.com (Y.Z.); zhanglih@gsau.edu.cn (L.Z.); 2 Gansu Key Laboratory of Animal Generational Physiology and Reproductive Regulation, Lanzhou 730070, China; 3 College of Animal Science and Technology, Gansu Agricultural University, Lanzhou 730070, China; dingziqiang1997@163.com

**Keywords:** food-derived melatonin, reproductive hormone, rumen microorganism, melatonin synthetase and receptors, melatonin fluctuations

## Abstract

In mammals, the melatonin (Mel) concentration in the gastrointestinal tract is 400 times greater than in the pineal gland. However, the origin of Mel in the gastrointestinal tract and its role in reproductive regulation remains unclear. Therefore, we analyzed three potential Mel sources (feed, microorganisms, and the rumen wall) for their contribution to high Mel levels in the rumen and their biological effects. The feed contained high Mel concentrations, and Mel in rumen fluid and blood peaked two hours after feeding. Rumen microbial analysis showed a strong positive correlation between Mel and specific microbes, including Megasphaera, Butyrivibrio, Acetobacter, and Olsenella. In vitro experiments indicated that rumen microorganisms synthesized Mel from tryptophan. The rumen wall also contains key enzymes, AANAT and HIOMT, which catalyze Mel synthesis and membrane receptors MT1 and MT2 that mediate the function of Mel, suggesting that the rumen wall synthesizes Mel. Mel peaked in both rumen fluid and blood two hours after feeding. Feeding also altered blood levels of Mel, Gonadotropin-releasing hormone (GnRH), Luteinizing hormone (LH), Follicle-stimulating hormone (FSH), progesterone (P4), and Estradiol (E2), with a correlation between Mel and fluctuations in GnRH, LH, P4, and E2 levels. Our findings suggest that feed is the primary source of high Mel levels in the rumen and impacts reproductive hormone fluctuations. This study elucidates the origin of high rumen Mel concentrations and reveals that food intake affects the natural secretion of various hormones, offering a new perspective on food sources for regulating reproductive physiology.

## Simple Summary

Melatonin is an indole hormone that is secreted by the pineal gland and has various biological functions, including circadian rhythm regulation, reproductive physiology and antioxidation. Melatonin concentration in the gastrointestinal tract far exceeds that found in the pineal gland, however, its source and potential effects remain unclear. This study used Small Tail Han sheep to examine the effects of feed, microorganisms, and the rumen wall on melatonin levels, as well as to measure changes and correlations between melatonin and reproductive hormone levels in the blood. These findings aim to elucidate the sources and potential functions of high melatonin concentrations in the rumen. The results provide a theoretical basis for understanding how food influences reproductive physiology.

## 1. Introduction

Melatonin (Mel) is an effective regulator of light-mediated physiological rhythms and is also involved in controlling inflammatory responses, oxidative stress, reproduction, and various signaling pathways [1,2]. Mel is predominantly synthesized in the pineal gland of mammals; however, it is also synthesized in the gastrointestinal tract [3,4,5]. Notably, Mel concentrations in the gastrointestinal tract are abnormally high (up to 400 times higher than levels in the pineal gland) and, while this phenomenon persists after pinealectomy, it diminishes with fasting [6,7]. Mel is abundantly present in various plants, Chinese herbal medicines, fruits, and fermented products. The enrichment of Mel in these sources improves plants’ tolerance to adverse environments and enhances their antioxidant and anti-aging properties [8,9]. Food-derived Mel can enter the bloodstream through gastrointestinal absorption, promoting sleep and antioxidant activity [8,9,10,11]. Research in mice and humans shows that fasting alters Mel levels in the gastrointestinal tract and blood circulation [12,13]. Another study found a transient increase in circulating Mel levels after meals, correlating with gastrointestinal Mel release and afternoon drowsiness [14]. Furthermore, gastrointestinal Mel concentrations in cattle and pigs fluctuate with feeding and vary significantly within the same intestinal segment, displaying different expression patterns [15]. These findings suggest that food intake is a key source of gastrointestinal Mel. However, limited information is available on Mel levels in rumen fluid and whether these are affected by feeding.

The rumen is a unique gastrointestinal structure in ruminants and is among the most efficient fermentation systems in nature. Its functionality depends on a highly complex microbial ecosystem, with up to 1 × 10^11^ microorganisms per milliliter of rumen fluid, including fungi, bacteria, and protozoa, with bacteria accounting for 95% of all microorganisms [16,17,18]. These microbes efficiently degrade fibers, synthesize vitamins and amino acids, and produce lactic acid and short-chain fatty acids (SCFAs), which fulfill 65–75% of the energy needs of ruminants [19,20]. Certain microorganisms (e.g., yeast and *Pseudomonas*) synthesize Mel or stimulate its production in animals and plants [9,21,22]. Mel modulates gastrointestinal microflora and circadian rhythm, contributes to inflammation control, and improves the intestinal microenvironment by regulating microbial diversity [23,24]. Mel also affects *Bifidobacterium* and *Desulfovibrio* abundance in the intestine, alleviating type 2 immune-related colitis via microbial-dependent mechanisms [25]. In mice studies, researchers found that Mel adjusted the levels of various microorganisms, restored microbial balance, reduced oxidative stress, and promoted gut health [26,27,28]. Additionally, Mel interacts with intestinal microbes, impacting the gut-brain and gut-liver axes. This helps regulate the development and outcomes of conditions such as obesity, insomnia, immunity issues, and metabolic disorders [29,30].

Mel plays an important role in regulating the neuroendocrine-reproductive axis. During pregnancy, it crosses the placenta and binds to its membrane receptors (MT1 and MT2) in fetal tissue, enhancing nutrient exchange and supporting vascular dynamics at the uterine-placental interface [31]. In a study on early pregnancy in mice, continuous oral administration of Mel reduced E2 and P4 secretion within 14 days and enhanced embryo implantation [32]. Mel also participates in the formation of the cumulus-oocyte complex and influences oocyte maturation; however, this regulatory effect diminishes after pinealectomy [33]. Human studies show that elevated nocturnal Mel levels in children inhibit GnRH, ovary function, and puberty development [34]. However, following Mel injection, ewe lambs and goat lambs showed earlier puberty onset and reproductive activity [35]. Additionally, intraperitoneal Mel injections significantly raised FSH and E2 levels, accelerating puberty onset in mice [36]. While the regulatory effects of Mel on reproductive hormones are well-documented, it remains unclear whether normal dietary intake (without supplementation) affects Mel levels in the rumen and blood, or its relationship with reproductive hormones.

This study aims to identify the Mel sources in the rumen of small-tail Han sheep and explore potential impacts through restricted feeding experiments and a subacute rumen acidosis (SARA) model. The findings offer new insights and data on the role of food-derived Mel in regulating the physiological functions of the body.

## 2. Materials and Methods

### 2.1. Animal Feeding and Management

All experimental procedures were conducted under the animal ethics regulations and guidelines of Gansu Agricultural University (approval number: GSAU-EthVMC-2021-007). A total of 15 small-tail Han sheep were housed at the animal farm of Gansu Agricultural University for this experiment. The sheep were approximately 5 months old, with an average body weight of 22.2 ± 2.35 kg. Before the experiment, the animals were fed for 14 days to acclimate them to the breeding environment and minimize stress-related impacts. Blood samples were collected from the animals for routine and biochemical tests, and regular deworming was carried out to maintain health. Feeding occurred at 08:00 and 20:00 daily at 2.7% of animal body weight. Concentrated feed and silage were mixed in specified proportions, with free access to drinking water and trace element licking bricks provided. The composition and nutritional components of the concentrated feed and silage are presented in Supplementary Tables S1 and S2. We conducted real-time monitoring of sheep signs and health status, collecting feed samples before and after the experiment and returning all samples to the laboratory for testing.

### 2.2. Sample Collection and Establishment of Subacute Rumen Acidosis (SARA) Model

Among the 15 female small-tail Han sheep (previously fed), 10 sheep of similar body weight were randomly assigned to a restricted feeding group (RF, *n* = 5) and a control feeding group (*n* = 5). At 0 h, 1 h, 2 h, 4 h, 6 h, 8 h, and 10 h post-feeding, 3 mL of blood (non-anticoagulant) was collected and centrifuged at 3000× *g* to obtain serum. Rumen fluid samples were collected through the oral cavity using an adult sheep esophageal vacuum pump at 0 h, 1 h, 2 h, and 4 h post-feeding to detect Mel. The fluid underwent static precipitation at 4 °C for 1 h, followed by centrifugation at 7500× *g* for 15 min; the supernatant was then collected, and this procedure was repeated three times. All samples were collected in a minimal light exposure environment.

At the end of the restricted feeding experiment, the experimental sheep were re-fed to achieve uniform health status. Subsequently, 15 small-tail Han sheep were randomly divided into a control group (*n* = 5), a SARA group (*n* = 5), and a treatment group (*n* = 5). To examine the impact of microbial changes on Mel levels in the rumen, the concentrate feed proportion was gradually increased to 80% of total feed, and feeding continued until the SARA model was established. The SARA establishment standard required a rumen pH of 5.2–5.6 for over 3 h daily, monitored dynamically over four weeks. Upon SARA onset, the feed of the treatment group was adjusted to match the ratio of the control group (20% concentrate feed), with NaHCO_3_ (5 g/d) added during the initial three treatment days. 

Finally, the animals in the control, SARA, and treatment groups were slaughtered. Before slaughter, sheep were fasted for 24 h and deprived of water for 12 h, then weighed. They were anesthetized with an intravenous injection of Jingsongling (2,4-dimethylaniline thiazole, 1 mg/kg). After full anesthesia, sheep were slaughtered, and rumen tissues along with other experimental samples were collected. Rumen fluid was collected for microbial diversity analysis, and dorsal and ventral sac tissues were obtained, washed with phosphate-buffered saline (PBS), and stored in liquid nitrogen for later experiments. Carefully cut segments of rumen tissue were fixed in tissue fixation and 2.5% glutaraldehyde solutions for morphological analysis.

### 2.3. Sample Pretreatment Before Determination

To determine the blood Mel levels, after gradient thawing and centrifuging at 5000× *g* for 15 min, the sample was filtered through a 0.45 μm pore membrane. Twenty mL of filtrate was extracted with 10 mL of dichloromethane (analytical purity), and the lower extract was collected; this process was repeated three times. After collection, the sample was precooled at −80 °C for over 24 h, freeze-dried (−40 °C, 48 h), dissolved in 1000 μL of methanol (chromatographic purity), vortexed for 45 s, and ultrasonicated for 20 min to ensure complete dissolution. An organic membrane with a 0.22 μm pore size was used to filter the dissolved solution, which was then tested directly or stored temporarily at −80 °C. The detection method for Mel levels in rumen fluid followed the blood method.

To confirm the Mel source in the rumen, we detected Mel levels in concentrated feed and silage. Briefly, 10 g of concentrated feed and silage were weighed into a 50 mL test tube, 10 mL of methanol was added, the sample was left to rest for 1 h, then ultrasonicated for 15 min and centrifuged at 10,000× *g* for 15 min at 4 °C. The supernatant was collected, filtered through a 0.22 μm filter, dried with nitrogen, dissolved in 1 mL of 80% methanol-water solution, oscillated for 15 min, filtered again using a 0.22 μm filter, and stored at −80 °C.

### 2.4. Hematoxylin and Eosin Staining

After removing the rumen tissue from the fixative, it was washed with running water, dehydrated by the alcohol gradient, cleared with xylene, embedded in wax, and sectioned into 5 mm thick paraffin slices. After dewaxing and antigen retrieval, hematoxylin was added for 2 min, washed with running water, differentiated with 1% hydrochloric acid-alcohol solution, stained with eosin for 45 s, sealed with gradient dehydration, and viewed under a microscope.

### 2.5. Transmission Electron Microscopy

The tissues of the rumen dorsal capsule and rumen abdominal sac in the control and SARA groups were fixed with 2.5% glutaraldehyde, then re-fixed with 1% osmium tetroxide. Acetone dehydration was performed in steps using a gradient of 30%→50%→70%→80%→90%→95%→100%. The sample was embedded in Ep812, and ultrathin sections measuring 60–90 nm were prepared using an ultrathin slicing machine. The sections were stained first with uranium acetate for 10–15 min, then with lead citrate for 1–2 min at room temperature. Prepared sections were finally observed and recorded using a transmission electron microscope (JEM-1400FLASH, JEOL, Tokyo, Japan).

### 2.6. Determination of GnRH, FSH, LH, P4, and E2 in Serum Using Enzyme-Linked Immunosorbent Assay

Following the enzyme-linked immunosorbent assay (ELISA) kit instructions, the concentrations of GnRH (gonadotropin-releasing hormone; ES69771, Yingxin, Shanghai, China), FSH (ES69987, Yingxin, Shanghai, China), LH (luteinizing hormone; ES70066, Yingxin, Shanghai, China), P4 (progesterone; ES69616, Yingxin, Shanghai, China), and E2 (estradiol; ES68558, Yingxin, Shanghai, China) in serum were measured under both feeding and restricted feeding conditions. Each sample was analyzed three times, and the OD value was detected at 450 nm within 15 min to ensure accuracy. The intra-plate and inter-plate coefficients of variation were below 10% and 15%, respectively. FSH and LH were expressed as mIU·mL^−1^, P4 as ng·mL^−1^, and GnRH and E2 as pg·mL^−1^.

### 2.7. High-Performance Liquid Chromatography and Liquid Chromatography-Mass Spectrometry

The concentration of Mel in rumen fluid, concentrated feed, and silage was measured using high-performance liquid chromatography (HP-LC). The HP-LC instrument (Agilent, Santa Clara, CA, USA) had a super binary gradient pump, thermostatic column, diode array detector, and ultraviolet detector. The mobile phases A, B, and C were ultra-pure water, methanol (chromatographic purity), and acetonitrile (chromatographic purity), respectively. Samples were passed through a C18 column (185 μm × 4 mm × 150 mm × 150 mm, Agilent, CA, USA) with a flow rate of 0.65 mL·min^−1^, an injection volume of 20 μL, column temperature of 30 °C, and detection wavelength of 233 nm. The elution gradient is shown in Supplementary Table S3.

Mel concentrations in the serum were analyzed by liquid chromatography-mass spectrometry (LC-MS). The instrument was a triple quadrupole mass spectrometer (1290-6460, Agilent, CA, USA) with a super-binary gradient pump, online vacuum degasser, automatic sampler, temperature-controlled column, and diode array detector. The mobile phase included 0.1% formic acid water (liquid A; chromatographic purity) and methanol (liquid B; chromatographic purity) with a flow rate of 0.3 mL·min^−1^. Each sample injection volume was 5 μL, with a column temperature of 40 °C. The Agilent C18 column (2.1 mm × 50 mm × 1.8 μm) was used in this study. The detail of the elution gradient is provided in Supplementary Table S3. The mass spectrometry conditions were as follows: positive ion mode, multiple reaction monitoring mode based on retention time to select parent ion size of 233.1 and daughter ion sizes of 174*/159 (with * as the quantitative ion), gas temperature of 350 °C, flow rate of 11 L·min^−1^, nebulizer pressure of 35 psi, and capillary voltage of 4000 V. Before injection, samples were flushed with methanol at a flow rate of 1 mL·min^−1^ to check pressure and balance.

### 2.8. Total RNA Extraction and RT-qPCR

Rumen tissue samples were treated with TRIzol (Solarbio, Beijing, China) for total RNA extraction. Absorbance at A260/280 nm was measured using a micro-nucleic acid analyzer, and RNA purity and integrity were confirmed by electrophoresis. RNA was reverse-transcribed into cDNA using a Prime Script RT Reagent Kit (TransGen Biotech, Beijing, China) with a gDNA Eraser. *AANAT*, *HIOMT*, *MT1*, and *MT2* in the rumen dorsal and ventral sac were quantified using *β-actin* as a reference gene. The conditions were set to 95 °C for 300 s, 95 °C for 5 s, and 60 °C for 30 s, repeating for 45 cycles. Results were calculated using the 2^−ΔΔCt^ method. Primer sequences are listed in Table 1.

### 2.9. Western Blotting

RIPA protein lysate (Solarbio) and protease inhibitor PMSF were added to the sample, which was centrifuged at 12,000× *g* and 4 °C for 15 min. Total protein was extracted, and the concentration was measured using the BCA protein detection kit (Solarbio). The protein supernatant was combined with a 5 × loading buffer, thoroughly mixed, denatured at 98 °C for 15 min, and stored at −80 °C. The laboratory-modified Western blotting technique was used to separate proteins using SDS-PAGE and proteins were then transferred to PVDF membranes. The membranes were rinsed with 1 × TBST and blocked with 5% skim milk powder in TBST for 1 h. Rabbit polyclonal antibodies, including AANAT (bs-3914R, 1:500, Bioss, Beijing, China), HIOMT (bs-6961P, 1:500), MT1 (bs-0027R, 1:500), and MT2 (bs-0963R, 1:500), with β-actin (bs-0061R, 1:4000) as the internal reference, were applied and incubated overnight at 4 °C. Afterward, the membrane was washed and treated with goat anti-rabbit secondary antibodies (bs-0295 G-HRP, Bioss) conjugated to horseradish peroxidase. Secondary antibody dilution was prepared at 1:4000 and incubated at 37 °C for 1 h. Immune complexes were visualized using enhanced chemiluminescence solution (Abnova, Taibei, Taiwan) and quantified with ImageJ software (Version number 1.8.0) .

### 2.10. Detection and Data Analysis of Rumen Microbial Diversity

The collected rumen fluid served as the source for nucleic acid extraction, using the TGuide S96 magnetic bead method soil/fecal genomic DNA extraction kit (Tiangen, Beijing, China). Nucleic acid concentration was assessed and amplified, followed by integrity detection of the amplified product. The Illumina Novaseq 6000 (Illumina, CA, USA) was used for double-end sequencing to construct a small fragment library and generate raw data. First, data quality was filtered using Trimmomatic (version 0.33), while primer sequences were identified and removed by Cutadapt (version 1.9.1). Then, USEARCH (version 10) was applied to splice double-end reads and remove chimeras (version 8.1). This resulted in high-quality sequences for further analysis. Species with an abundance >1% were annotated through the Silva annotation database (Release132, http://www.arb-silva.de, accessed on 19 May 2024). Alpha-diversity and beta-diversity were evaluated with QIIME and visualized using R software (Version 2.15.3).

### 2.11. Culture of Rumen Microorganisms In Vitro

A rumen buffer was prepared with 9.8 g NaHCO_3_, 9.3 g NaHPO_4_·7H_2_O, 0.47 g NaCl, 0.57 g KCl, 0.12 g MgSO_4_·7H_2_O, 0.04 g CaCl_2_·2H_2_O, 1.25 mg Resazurin, 0.015 g MnCl_2_·4H_2_O, 0.002 g CoCl_2_·6H_2_O, and 0.012 g FeCl_3_·6H_2_O. The buffer solution was dissolved in a thermo-static shaker at 39–40 °C, with CO_2_ introduced and the pH adjusted to 6.8–7.0. Rumen fluid (control group and SARA group) was collected and mixed with artificial saliva at a 1:1 ratio, with CO_2_ infused continuously. We accurately measured 2.5 mg of tryptophan and 5 g of concentrated feed and added these to the mixed rumen fluid, with three replicates for each group. The fluid was cultured in an artificial rumen device for 6 h, with 10 mL samples collected at 0 h, 0.5 h, 1 h, 2 h, 4 h, and 6 h to detect the melatonin concentration. To minimize undigested feed effects on the in vitro rumen fluid culture, the animals were restricted from eating for half a day, and filtered rumen fluid was collected.

### 2.12. Statistical Analysis

All experimental data are represented as the mean ± standard error (mean ± SEM). SPSS software (23.0, IBM, Armonk, NY, USA) was used for the analysis. Data were tested for normal distribution and covariance, and one-way ANOVA was followed by Duncan’s new multiple-range test. A *p*-Value of <0.05 was considered significant. A correlation coefficient (r) < 0 or >0 was interpreted as a negative or positive correlation, respectively; the closer was to −1 or 1, the stronger the correlation.

## 3. Results

### 3.1. Melatonin Content Changes in Feed and Rumen

**Figure 1:**
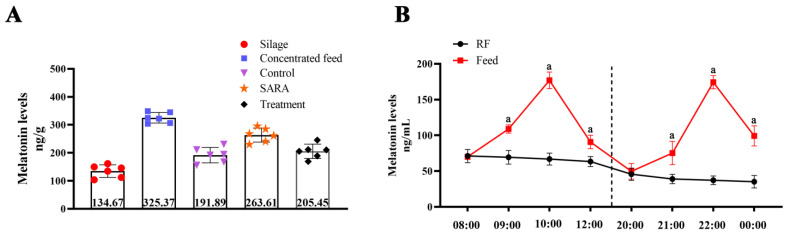
Changes in Mel contents in feed and rumen. (**A**) HP-LC detection of Mel content in different feed groups. (**B**) Changes in rumen Mel concentration over time within 4 h after feeding in the control group. ‘a’ indicates a significant difference between the feed and RF group in the same period (*p* < 0.05). RF: restricted feeding group.

The results showed that Mel concentrations in silage, concentrated feed, control group feed, SARA group feed, and treatment group were 134.67 ± 9.17 ng·g^−1^, 325.37 ± 7.69 ng·g^−1^, 191.89 ± 11.19 ng·g^−1^, 263.61 ± 10.26 ng·g^−1^, and 205.45 ± 10.39 ng·g^−1^, respectively (Figure 1A). After feeding, Mel concentration in the rumen fluid was significantly higher than that in the rumen fluid, peaking 2 h after feeding (Figure 1B).

### 3.2. The Establishment and Verification of the SARA Model

**Figure 2:**
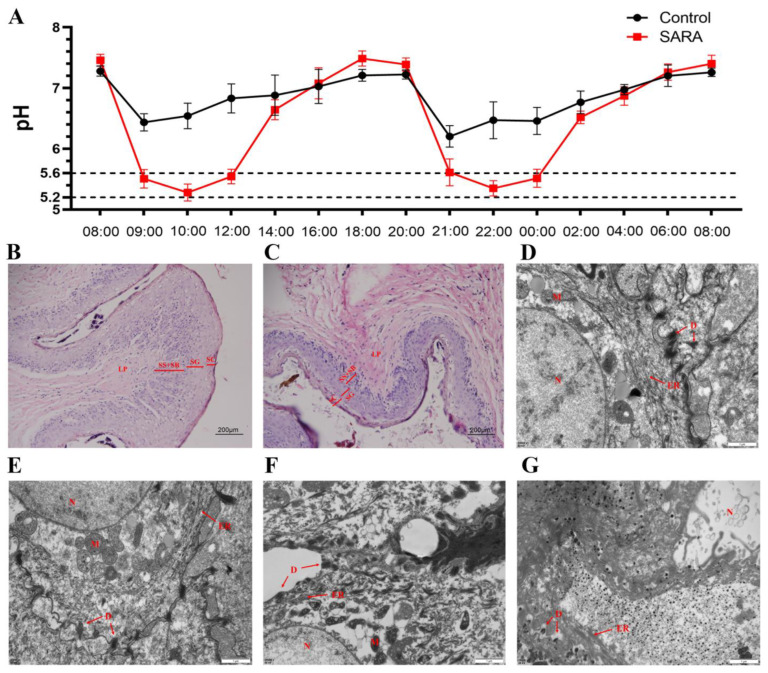
Rumen pH detection and histomorphological observations. (**A**) Rumen pH detection over a period of 24 h. (**B**) Control rumen epithelium. (**C**) The rumen epithelium of the SARA group. (**D**) The spinous layer cells of the ventral sac in the control group were observed under an electron microscope. (**E**) The dorsal sac spinous layer cells of the control group. (**F**) The rumen ventral sac spinous layer cells of the SARA group. (**G**) The rumen dorsal sac spinous layer cells of the SARA group. SC: stratum corneum; SG: stratum granulosum; SS: stratum spinosum; SB: stratum basale; LP: lamina propria; D: desmosomes; M: mitochondria; N: nucleus; ER: endoplasmic reticulum.

To verify relationships between rumen microorganisms and Mel, the SARA model of small-tail Han sheep was established. The results showed that, over 24 h, rumen pH dropped to between 5.2 and 5.6 for two periods exceeding 3 h (Figure 2A). Hematoxylin and eosin staining indicated that, compared to the control group, rumen epithelial cells in the SARA group appeared loose and irregular, with significant thickening of the stratum corneum and reduced thicknesses of the granular, spinous, and basal layers (Figure 2B,C). Electron microscopy revealed that the nucleus, endoplasmic reticulum, desmosomes, and mitochondria of the control group were intact, well-defined, and exhibited clear boundaries with small intercellular spaces (Figure 2D,E). Conversely, cell boundaries in the SARA group appeared blurred, vacuoles were present, desmosome junctions disappeared, and the structure was degraded, making organelles difficult to identify (Figure 2F,G). These observations confirmed that the SARA model of small-tail Han sheep was successfully established.

### 3.3. Changes in Rumen Microflora and Analysis of Melatonin Relationship with Microorganisms 

**Figure 3:**
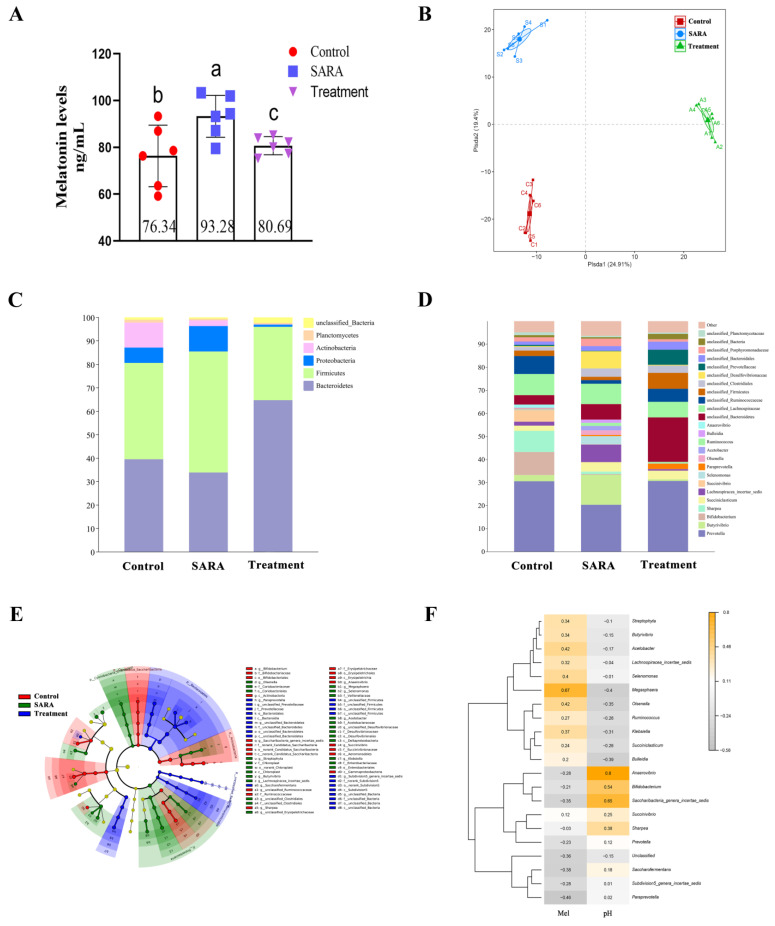
Correlation analysis of Mel content, pH, and microorganisms in the rumen fluid. (**A**) Concentration of Mel in rumen fluid. (**B**) PLS-DA cluster analysis among groups. (**C**) The microbial abundance of each group at the phylum level. (**D**) The microbial abundance of each group at the genus level. (**E**) Analysis of the difference in LEfSe between groups. (**F**) Heat map of correlation analysis between Mel, pH, and microorganisms (genus level). Different letters indicate significant differences between groups (*p* < 0.05).

Figure 3 shows the Mel concentration and microflora changes in rumen fluid across different groups. The results indicated that Mel concentrations in rumen fluids of the control, SARA, and treatment groups were 76.34 ± 5.36 ng·mL^−1^, 93.28 ± 3.67 ng·mL^−1^, and 80.69 ± 1.57 ng·mL^−1^, respectively (Figure 3A). After SARA, microbial flora in the rumen was analyzed using 16S rRNA. PLS-DA classification showed no significant clustering, but intergroup differences were significant (Figure 3B). Phylum-level analysis indicated that Firmicutes and Proteobacteria abundances were higher in the SARA group than in the control group, while Actinobacteria abundance was significantly lower. In the treatment group, Bacteroidetes abundance significantly increased, while Proteobacteria abundance significantly decreased (Figure 3C). Genus-level analysis revealed that *Butyrivibrio*, *Lachnospiracea_incertae_sedis*, *Selenomonas*, *Olsenella*, unclassified_Clostridiales, and unclassified_Desulfovibrionaceae abundances were higher in the SARA group than in the control group, while *Prevotella*, *Bifidobacterium*, *Sharpea*, *Succinivibrio*, and unclassified_Ruminococcaceae abundances were lower. In the treatment group, *Paraprevotella*, unclassified_Bacteroidetes, unclassified_Firmicutes, and unclassified_Prevotellaceae abundances were significantly higher than in the control group, whereas *Bifidobacterium*, *Sharpea*, and *Succinivibrio* abundances were significantly lower (Figure 3D). LEfSe analysis screened marker LDA with significant differences (LDA > 3.5). The results indicated that the most significant genera in the control, SARA, and treatment groups were *Bifidobacterium*, *Butyrivibrio*, and unclassified_Bacteroidetes, respectively (Figure 3E, Supplementary Table S4). Correlation analysis of microorganisms with Mel and pH showed that Mel was highly positively correlated with *Megasphaera*, *Butyrivibrio*, *Acetobacter*, and *Olsenella*, while pH was highly positively correlated with *Anaerovibrio*, *saccharibacteria_genera_incertae_sedis*, and *Bifidobacterium* (Figure 3F).

### 3.4. In Vitro Culture of Rumen Microorganisms 

**Figure 4:**
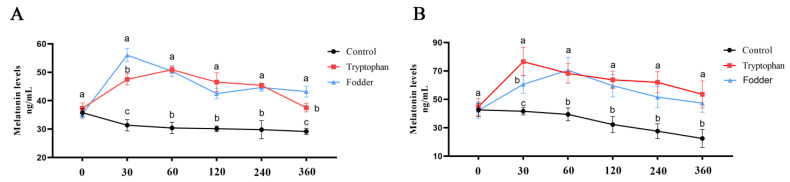
Effects of tryptophan supplementation and feed on Mel in artificial rumen. (**A**) Mel concentration in rumen fermentation tank of the control group. (**B**) SARA group. Control group was not supplemented with feed or tryptophan. Different letters indicate significant differences between groups (*p* < 0.05).

To further investigate the relationship between rumen microorganisms and Mel, we cultured rumen microorganisms from the control (Figure 4A) and SARA (Figure 4B) groups in vitro using an artificial rumen technique. Tryptophan was metabolized by rumen microorganisms to produce Mel. The rumen fermentor in the SARA group demonstrated higher tryptophan utilization efficiency and elevated Mel concentrations.

### 3.5. Expression of Melatonin Synthetases and Its Receptors in Rumen Wall

**Figure 5:**
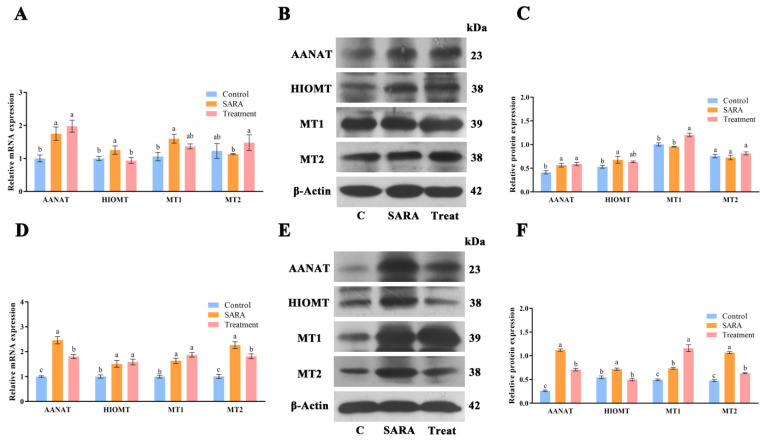
Expression of Mel synthetases and receptors in rumen dorsal sac and abdominal sac. (**A**) Relative mRNA levels of Mel synthetases (AANAT, HIOMT) and Mel receptors (MT1, MT2) in rumen dorsal sac. (**B**) Mel synthetase and receptor protein immunoblotting in the dorsal sac. (**C**) Relative protein expression levels of Mel synthetases and receptors in the dorsal sac. (**D**) Relative Mel synthetase and receptor mRNA levels in rumen ventral sac. (**E**) Immunoblotting of Mel synthetases and receptor proteins in the ventral sac. (**F**) Relative protein expression levels of Mel synthetases and receptors in the ventral sac. Different letters indicate significant differences between groups (*p* < 0.05).

To examine Mel synthesis in the rumen wall, we measured the expression of Mel synthetases AANAT and HIOMT, as well as Mel receptors MT1 and MT2 in the rumen dorsal and ventral sacs. In the rumen dorsal sac, AANAT mRNA and protein expression in the SARA and treatment groups were significantly higher than in the control group (*p* < 0.05). HIOMT mRNA expression in the SARA group exceeded that of the treatment and control groups, with its protein expression also higher than in the control group (*p* < 0.05). *MT1* mRNA levels in the control group were significantly lower than in the SARA group, with MT1 protein expression lower than in the treatment group (*p* < 0.05). MT2 mRNA and protein levels did not differ significantly between the control, SARA, and treatment groups (Figure 5A–C). 

In the rumen ventral sac, AANAT mRNA and protein expression in the SARA group were significantly higher than in the control and treatment groups. *HIOMT* mRNA levels in the SARA and treatment groups exceeded those in the control group, and HIOMT protein expression in the SARA group was significantly higher than in the control and treatment groups (*p* < 0.05). MT1 mRNA and protein levels in the control group were significantly lower than in the SARA and treatment groups. MT2 mRNA and protein expression in the SARA group were significantly higher than in the control and treatment groups (Figure 5D–F).

### 3.6. Fluctuation in Melatonin and Reproduction-Related Hormones After Feeding

**Figure 6:**
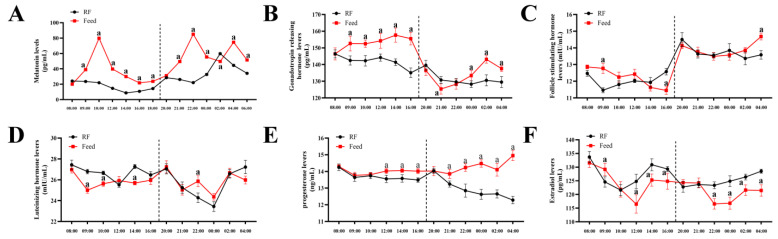
Changes in the blood concentration of Mel, GnRH, FSH, LH, P4, and E2 after feeding. (**A**) Mel concentration changes in the blood after feeding during the day and night. (**B**) GnRH. (**C**) FSH. (**D**) LH. (**E**) P4. (**F**) ‘a’ indicates significance at *p* < 0.05. E2. RF denotes under restricted feeding conditions; RF: restricted feeding group. The vertical line represents the time boundary between morning and afternoon testing on the same day.

As noted in Result Section 3.1, Mel concentration in the rumen peaked at 191.89 ng/mL after 2 h of feeding, approximately three times higher than under restricted feeding. To assess the potential impact on other reproductive hormones, we measured Mel, GnRH, FSH, LH, P4, and E2 concentrations in the blood following feeding and feeding restriction. The results showed three Mel concentration peaks in the blood, occurring 2 h after morning feeding and 2 and 6 h after evening feeding (Figure 6A). The timing of the first two peaks was highly consistent with Mel levels in the rumen. Compared to restricted feeding, feeding induced fluctuations in blood GnRH, FSH, LH, P4, and E2 levels (Figure 6B–F).

### 3.7. Correlation Between Feeding and Restricted Feeding on Hormones in Blood

**Figure 7:**
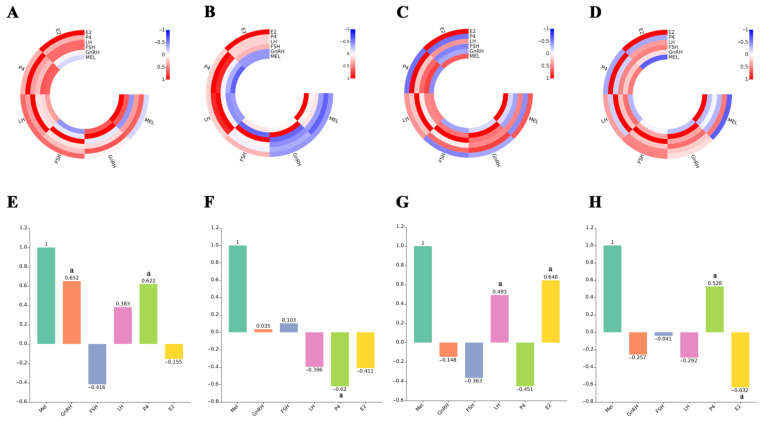
Correlation analysis between Mel, GnRH, FSH, LH, P4, and E2 in blood. (**A**,**B**) Daytime restricted feeding group; (**C**,**D**) daytime feeding group; (**E**,**F**) nighttime restricted feeding group; (**G**,**H**) nighttime feeding group. ‘a’ indicates significance at *p* < 0.05, and different values indicate a correlation between Mel and reproductive hormones.

To further explore the correlation between fluctuations in reproductive hormones and Mel, we performed a correlation analysis of Mel, GnRH, FSH, LH, P4, and E2 (Figure 7A–H). Following day or night feeding, hormone correlations shifted (Figure 7A–D). Additionally, we found a positive correlation between Mel and GnRH and P4 in the daytime restricted feeding group (Figure 7E), a negative correlation between Mel and P4 in the daytime feeding group (Figure 7F), a positive correlation between Mel and LH and E2 in the nighttime restricted feeding group (Figure 7G), and a positive correlation between Mel and P4 but a negative correlation between Mel and E2 in the nighttime feeding group (Figure 7H).

## 4. Discussion

Our experiment results confirmed that Mel levels in the rumen were 2000 times higher than in the blood of sheep, originating from feed, rumen microbial metabolism, and rumen wall synthesis. The diet used in this experiment, particularly the concentrated feed, contained high Mel levels, similar to those in other plants like licorice, rice, and soybeans, and Mel levels exceeded those found in animal serum [37,38]. Mel in the rumen fluid and blood peaked two hours after feeding, indicating that Mel in the blood is absorbed from the diet via the gastrointestinal tract. Although Mel peak timing differed, the results aligned with findings for pigs, rats, and humans [11,39,40]. This may relate to the distinct gastric structure and bioavailability in ruminants. Notably, three Mel peaks occurred in the blood after night feeding. The first two peaks were synchronized with feeding, while the third peak matched the reported pineal gland secretion from 2 a.m. to 4 a.m. This pattern may involve the master clock and secondary clock, where the master clock (regulated by the light-dark cycle) is somewhat unaffected by meal-time synchronization effects [41,42].

The SARA model identified the relationship between the microbial community and Mel. In this study, we observed different degrees of damage to the rumen epithelium, blurred cell boundaries, and mitochondrial degradation after SARA occurred, aligning with studies in yaks and goats [43,44]. The level of Mel in the SARA group rose significantly. LDA effect size and correlation analysis indicated that the abundance of *Megasphaera elsdenii*, *Butyrivibrio*, *Acetobacter*, and *Olsenella* notably increased at the genus level, showing a strong positive correlation with Mel. The shift in this microbiota resembled that observed in SARA dairy cows [45,46]. *Megasphaera elsdenii* uses lactic acid to produce butyric acid, an SCFA that aids in reducing oxidative stress, immune defense, and inflammation in humans, acting in synergy with Mel [47,48,49]. Additionally, SCFAs bind to apical membrane receptors on the cell surface, raising the content of the Mel precursor 5-HT [50]. *Butyrivibrio* is viewed as a potentially beneficial bacterium. Night fasting experiments indicate that *Butyrivibrio* not only produces butyric acid but also exhibits a clear circadian rhythm [51]. Studies in boars have shown a significant positive correlation between *Butyrivibrio*, Mel, and amino acids, benefiting sperm quality [52]. In an intestinal metabolism experiment, *Acetobacter* produced and utilized the Mel precursor tryptophan, and also decomposed various amino acids, including leucine and valine, to generate SCFAs [53]. *Acetobacter* also influences egg development and ovarian reproductive potential in *Drosophila melanogaster* [54]. *Olsenella* produces SCFAs using amino acids, playing a role in immune regulation and inflammatory response [55,56]. In vitro culture experiments revealed that rumen microorganisms synthesize Mel from tryptophan, increasing Mel concentration in the fermentation broth, with a higher tryptophan utilization rate in the SARA group. This result is consistent with the increase in Mel-related microorganisms in the SARA group. While we observed a correlation between these microorganisms and Mel, previous studies reported that these microorganisms directly or indirectly perform functions similar to those of Mel. However, further studies are necessary to understand the relationships between Mel and these microorganisms.

The rumen wall is also an important source of Mel in the rumen. Our results showed that the rumen wall contains not only AANAT and HIOMT, the key synthases of Mel, but also Mel membrane receptors. This suggests that the rumen wall synthesizes Mel and serves as a target organ. Following SARA, Mel synthase levels significantly increased, potentially related to antioxidative and anti-inflammatory roles [57]. Furthermore, the increase in Mel may regulate the function of rumen microflora and play a synergistic role in anti-inflammation and epithelial barrier protection [25,28].

Previous studies have shown that eating influences blood Mel levels, Mel circadian rhythms, intestinal microbial composition, and glucose tolerance, suggesting a possible relationship between these effects and human and animal health [42,51]. However, this study is the first to reveal a potential connection between dietary Mel and reproductive physiology. Specifically, we found a significant correlation between post-feeding fluctuations in Mel levels and concurrent changes in GnRH, LH, P4, and E2 in circulating blood. Mel, an amphiphilic molecule, crosses the blood-brain barrier, allowing dietary Mel to potentially regulate the hypothalamic-pituitary-gonadal axis [58]. Studies on Mel and GnRH have shown that Mel upregulates RF-amide-related peptide (RFRP) and inhibits GnRH synthesis in the hypothalamus by affecting the kisspeptin/RFRP systems, which could influence puberty [59]. Mel also modulates nerve signal transduction via vascular endothelial growth factor A/B (VEGF-A/B), regulates GnRH secretion, and impacts the reproductive development of sheep [60]. Injecting Mel in late spring results in earlier puberty and reproductive activity in well-fed ewes [61]. However, early puberty requires sufficient weight (50–70% of adult sheep weight) because physiologically active tissues, such as fat and muscle, interact through endocrine and reproductive axes [62,63]. Polkowska et al. [64] found that female lambs are more sensitive to photoperiod changes, potentially linked to puberty onset and GnRH and LH secretion. We hypothesize that diet may regulate animal reproductive physiology, possibly involving Mel. Further research should investigate changes in melatonin levels in blood and cerebrospinal fluid near the ovary. Additionally, exploring the effects of dietary Mel may significantly enhance livestock reproduction.

## 5. Conclusions

Mel in the rumen originates from feed decomposition, microbial metabolism, and rumen wall synthesis, with feed decomposition as the primary factor causing rumen Mel fluctuations. Animal feed contains abundant Mel, potentially serving as a major source of Mel in blood and affecting GnRH, LH, P4, and E2 levels, thus influencing puberty and reproductive physiology. This study clarifies the origins of high Mel concentrations in the rumen and offers new insights and data on the potential for reproductive physiology regulation via Mel from food sources.

## Figures and Tables

**Table 1:** Primer sequences of target and housekeeping genes.

Genes	Primer Sequences (5′→3′)	Length (bp)	Accession No.
*AANAT*	F: CCCCCTGAATCTGGACGAG R: CACAGGGAGCCGATGATGAAGG	141	NM_001009461.1
*HIOMT*	F: GCTCTTTATGCTCAGAAGGACTCAA R: ACAAGCTGATGGAACAGAGAACTG	107	NM_001306120.1
*MT1*	F: AGCACGAATTCCCTCTGCTA R: GAGCATCGGAACGATGAAAT	183	NM_001009725.1
*MT2*	F: AGGTCAAGGCGGAGAGCAA R: GCCACTTCTTCGGGGTCAA	148	NM_001130938.1
*β-actin*	F: CCATTGAGCACGGCATTGT R: GCAGGGGTGTTGAAGGTCTC	184	U39357.2

## Data Availability

The datasets produced and/or analyzed during the current study are available from the corresponding author upon reasonable request.

## Supplementary Materials

The supporting information is downloadable at https://www.mdpi.com/article/10.3390/ani14233451/s1, Supplementary Table S1: Composition and Nutritional Components of Concentrated Feed (Dry Matter Basis); Supplementary Table S2.: Nutrients of Silage Feed; Supplementary Table S3: Elution gradient of high-performance liquid chromatography and liquid chromatography-mass spectrometry; Supplementary Table S4: Microbial LDA values were different among the groups.
