# Supplementary material for: Melatonin Sources in Sheep Rumen and Its Role in Reproductive Physiology
Source: Animals (Basel). 2024 Nov 28;14(23):3451. doi: 10.3390/ani14233451 (PMC11640204; doi:10.3390/ani14233451)
Supplement: Supplementary file 1 [file animals-14-03451-s001.zip › Supplementary Table S1.pdf]

Table S1 Composition and Nutritional Components of Concentrated Feed (Dry Matter Basis)

Items	Content, %
Corn silage	35.1
Alfalfa hay	17.2
Barley	23.6
Low erucic acid rapeseed meal	7.5
Soybean meal	8.3
Corn gluten meal	2.0
Corn distillate	2.5
Premix	2.1
Energy enhancer	1.2
Mineral salt	0.5
Total	100
**Nutritional levels**	
Digestible Energy, MJ/kg	15.57
Crude protein, %	13.30
Crude fat, %	2.0
Calcium, %	2.4
Phosphorus, %	0.55
Crude ash, %	6.2
Neutral detergent fiber, %	54.6
Acid detergent fiber, %	29.6

Premix provides: Zn 7.5g, Fe 0.9g, Cu 1.5g, Mn 1g, I 0.1g, Co 20mg, VE 1.2g, VA 270 000IU, VD 318 000IU. Digestive energy is a calculated value, while other results are measured values.
