# Supplementary material for: Melatonin Sources in Sheep Rumen and Its Role in Reproductive Physiology
Source: Animals (Basel). 2024 Nov 28;14(23):3451. doi: 10.3390/ani14233451 (PMC11640204; doi:10.3390/ani14233451)
Supplement: Supplementary file 1 [file animals-14-03451-s001.zip › Supplementary Table S2.pdf]

Table S2 Nutrients of Silage Feed

Items	Nutritional levels
Crude protein, %	8.3
Crude fat, %	2.3
Calcium, %	0.32
Phosphorus, %	0.17
Crude ash, %	6.7
Neutral detergent fiber, %	49.5
Acid detergent fiber, %	28.0
Fresh sample moisture, %	66.0
Air-dried sample moisture, %	4.3
